# Clinical and Functional Characteristics of a New Phenotype of SMA Type I among a National Sample of Spanish Children: A Cross-Sectional Study

**DOI:** 10.3390/children10050892

**Published:** 2023-05-16

**Authors:** Beatriz de-Andrés-Beltrán, Javier Güeita-Rodríguez, Domingo Palacios-Ceña, Ángel Luis Rodríguez-Fernández

**Affiliations:** 1 Department of Physical Therapy, Centro RIE (Rehabilitación Infantil Especializada), 28050 Madrid, Spain; bdeandres@centrorie.com; 2 International Doctorate School, Rey Juan Carlos University, 28008 Madrid, Spain; 3 Department of Physical Therapy, Occupational Therapy, Physical Medicine and Rehabilitation, Research Group of Humanities and Qualitative Research in Health Science, Rey Juan Carlos University, 28922 Alcorcón, Spain; domingo.palacios@urjc.es; 4 Department of Physical Therapy, Universidad San Pablo-CEU, CEU-Universities, 28925 Alcorcón, Spain; alrodfer@ceu.es

**Keywords:** Spinal Muscular Atrophy type I, physiotherapy, scoliosis, motor function, rehabilitation

## Abstract

Spinal Muscular Atrophy (SMA) type I has classically presented extremely severe clinical features. New pharmacological treatments have led to a new phenotype of SMA. The aim of this study was to describe the current health and functional status of children with SMA. A cross-sectional study was conducted based on the STROBE guidelines. Patient questionnaires and standardized tools were used. A descriptive analysis was conducted establishing the proportions of subjects for each of the characteristics of interest. In total, 51 genetically confirmed SMA type I subjects were included. Fifty-seven percent received oral feeding, 33% received tube feeding and 10% combined both. Moreover, 21.6% had tracheostomies, and 9.8% needed more than 16 h/d ventilatory support. Regarding orthopedic status, 66.7% had scoliosis, and 68.6% had hip subluxation or dislocation. Up to 67% were able to sit independently, 23.5% walked with support and one child walked independently. Current SMA type I is a different entity from the classic phenotype but also from types II and III. In addition, no differences were found between SMA type I subgroups. These findings may enable the professionals involved in the care of these patients to improve their interventions in terms of prevention and rehabilitation measures for these children.

## 1. Introduction

The term Spinal Muscular Atrophy (SMA) refers to a series of genetic disorders characterized by degeneration of the cells of the anterior horn of the spinal cord [1,2,3]. It is the second most frequent cause of death due to autosomal recessive disease after cystic fibrosis [1,4], and the first in infancy [5]. In the “classic” clinical description of SMA, patients with SMA type I (or Werdnig and Hoffmann disease) are described as having hypotonia, symmetrical muscle weakness, poor head control, and decreased or even absent reflexes before six months of age [2,4,6]. Weakness of the intercostal muscles, with relative maintenance of the diaphragm, causes a bell-shaped thorax accompanied by paradoxical breathing [2,6]. These children have weakness of the tongue and swallowing muscles, developing a risk of aspiration and respiratory failure before the age of two years [1,6,7,8]. Most are button fed (before 24 months of age), and many require intervention for gastroesophageal reflux. Those who survive the first few years have bilateral subluxation or dislocation of the hips, and by one year of age, they develop scoliosis [9]. The loss of motor capacity is described as between 1.27 points [10] and 10.7 points per year [11] in the specific assessment scales.

The current classification subdivides patients with SMA type I into type Ia (symptom onset before one month), type Ib (symptom onset between one month and three months) and type Ic (symptom onset between three and six months) [12], it has been reported that early onset of symptoms carries a worse prognosis, with type Ia patients having a more severe phenotype [13]. This is the most frequent type of SMA (about 60% of cases) [3,4], although there is very little literature on the subject due to their premature death. When the classification was developed in the 1990s, the survival probability of these children at 2, 4, 10, and 20 years was 32%, 18%, 8%, and 0%, respectively [8], even lower in other cohorts [7], placing the mean age of death at 9.6 months of age. However, since the description of the pathology and the development of these studies in the 1990s and until the appearance of the first medication to treat SMA in 2017, there was an evolution in terms of nutritional and respiratory care that led to an increase in the mean age of survival [10,14], to the extent of completely changing the survival rates: 50%, 40%, 30%, 30%, 30% and 30% at 1, 2, 4, 10 and 20 years, respectively [15].

There are currently three therapeutic options available for SMA patients that have been developed based on the understanding of the molecular basis of SMA (Nusinersen, Onasemnogene abeparvovec and Risdiplam) and which focus on *SMN1* gene modification or replacement or RNA modulation [16]. Advances in clinical trials have led to clinical improvements that are completely changing the therapeutic approach to these patients [9,12,16,17]. Nonetheless, it should be noted that despite this change or evolution in the phenotype of the disease, some treated patients may be non-responders, slow responders or begin to respond after many months of treatment [18].

The health alterations that accompany this pathology have been described when no treatment is applied to change the natural history of the disease; mainly at the respiratory and nutritional levels [7,10,13,19], but also at the cardiac or endocrine level [9], although less information is available on its orthopedic development [20]. In addition, there are references regarding the evolution of patients who present milder versions of the disease (SMA type II and III), including orthopedic information [9,21,22,23]. However, it is possible that a patient with SMA type I who has received treatment, by attaining the functional capacity of a patient with SMA type II or III, may have similar respiratory or orthopedic alterations. The new functional capacities of these patients are being recorded in the clinical trials of pharmacological treatments, however, the status of the secondary alterations that accompany the pathology, both respiratory, nutritional, and orthopedic alterations and needs, have not been described. The survival of these patients opens a new path for which reliable data are needed and it is, therefore, essential to monitor and record the status of children with SMA, both those who have undergone the new pharmacological therapies and those who have not, so that their treatment and care can be improved [24] and to optimize their quality of life and participation in society.

Consequently, a new spectrum of the disease is expected in the coming years, and therefore it is necessary to better understand the evolution of the associated alterations, such as scoliosis or hip dislocation, dysphagia or reflux in treated patients who are developing functional capacities superior to those classically achieved by patients with SMA type I. This will allow us to discern whether the prevalence of these associated alterations is due not only to motor capacity but also to the phenotype of the disease itself and to predict how these will affect children’s functionality and participation in society. The aim of this study was to describe the functional and health status of children with SMA type I registered in Spain. Specifically, by comparing the functional and health status of children with SMA type I according to the age of symptom onset and therefore their classification subgroup.

## 2. Materials and Methods

### 2.1. Study Design

**Figure 1:**
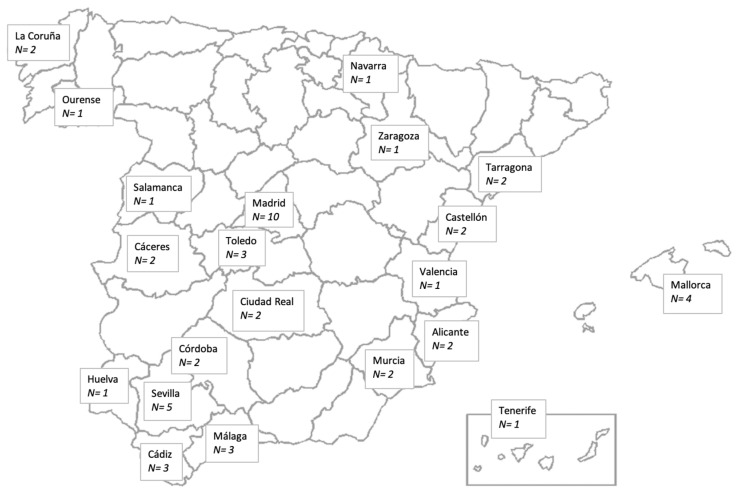
Geographical distribution of the sample.

A descriptive and cross-sectional observational study was carried out, including SMA patients distributed in 21 provinces in Spain, and centrally coordinated by the Rey Juan Carlos University of Madrid (Figure 1).

The STROBE guidelines for reporting cross-sectional studies were followed [25] (see checklist in Supplementary Materials Table S1).

### 2.2. Participants and Recruitment

A convenience sampling was performed, reaching 85% of the entire population of SMA type I in Spain. The Fundación de familias y afectados de AME de España (FundAME), was contacted, which has a national registry program of SMA patients (RegistrAME), which includes almost all the cases in Spain (https://wacean.com/registroame/ consulted on November 2021). All registered families (*n* = 60) were contacted and offered to participate in this study in December 2021. Interested families (*n* = 52) contacted the principal investigator (B.d.A.), who conducted face-to-face visits to all participants between March 2022 and February 2023. Given the special frailty of these patients, it was determined that the best way to assess them was for the researcher to visit them in their natural environment. Initially, 52 subjects were included, although one subject could not be visited for family reasons. Even so, the high coverage rate (85% of the population) ensured a high representativeness of the sample studied and correct control of possible biases.

The only conditions for an appointment were to be living patients with a confirmed genetic diagnosis of SMA type I, as well as having informed parental consent.

### 2.3. Data Collection

Data collection was carried out through two components: the administration of a structured self-created questionnaire based on literature reviews, which was completed by the primary caregivers. Also, by administering standardized scales chosen according to their capacities and age, to assess motor ability, upper limb function and motor milestones reached.

#### 2.3.1. Sociodemographic and Physical Conditions

Information on the sociodemographic status and physical condition was obtained using a 29-item questionnaire, which explored categories such as the age of disease onset, diagnosis, treatment, whether they had received surgery or experienced fractures, type of feeding, ventilatory support, orthopedic alterations, technical aids and therapies received.

#### 2.3.2. Functional Assessments

The Children’s Hospital of Philadelphia Infant Test of Neuromuscular Disorders (Chop-Intend): [26,27] This scale was developed in 2010 to assess patients with significant weakness, and great difficulty performing anti-gravity movements. This scale has been shown to have adequate reliability values, to be easy to administer and is well tolerated by children with neuromuscular conditions presenting with extreme weakness; moreover, it is the scale used in both clinical follow-ups and clinical trials to assess evolution. Its interobserver reliability is very high (interclass correlation ratio of 0.98) with an intraobserver reliability of 0.96; it also has excellent internal consistency (>0.9) [26].

The Hammersmith Infant Neurological Examination, Section 2 (HINE-2): [28,29] In order to assess the motor development of the smallest and/or weakest patients, different studies have been carried out with Section 2 of the HINE, where eight motor milestones of the motor development of children between 2 and 24 months of age are assessed, and this section of the scale has been adopted as a standard assessment both in the clinic and in trials with these patients [17,30].

The Hammersmith Functional Motor Scale Expanded (HFMSE): [31] This scale measures the motor ability of children who are capable of greater motor performance than children classically classified as having SMA type I. It assesses their sitting, turning, crawling, standing, kneeling, jumping, and stair-climbing abilities. It has high reliability (interclass correlation ratio for interobserver reliability of 0.99) and its validity was established by correlation with the Gross Motor Function Measure, observing an almost perfect correlation (r = 0.98) [32]. This version of the scale is currently the most widely used and the one used in clinical trials and is valid from the age of 24 months for both ambulatory and non-ambulatory patients [33].

The Revised Upper Limb Module (RULM): [34] This scale assesses upper limb function. It is currently used in clinical practice and in clinical trials, with good levels of reliability (excellent interobserver reliability of 0.93) and validity and is used in children from 30 months of age to adulthood, even in the weakest children who cannot be assessed for gross motor function [34]. The administration time of this scale is between 5 and 20 min [34].

### 2.4. Analysis of Results

Although the sample studied was 85% of the population diagnosed with SMA in Spain, given the overall size of the sample and the subsequent segmentations into the different subgroups, a nonparametric statistical analysis was performed. A descriptive analysis was carried out by establishing proportions in the case of qualitative variables, and medians (interquartile range) in the case of quantitative variables. To evaluate the association between the different qualitative variables and the subgroups of children according to the time of symptom onset (SMA type Ia, Ib and Ic), Chi-square tests were used. A comparison of different quantitative variables between these three classification subgroups was performed using a Kruskall-Wallis test. Values of *p* < 0.05 were considered significant.

### 2.5. Ethical Considerations

The study was approved by the Ethics Committee of Rey Juan Carlos University (3005202114821, 21 July 2021). Written informed consent was obtained from the parents of all study participants. The study was performed in accordance with the approved guidelines and regulations of the participating institution.

## 3. Results

### 3.1. Patient Characteristics and Demographics

Fifty-one patients (20 girls, 31 boys) were visited in person at their homes, with a mean ± SD age of 3.7 ± 2.5 years (Table 1). The youngest patient was three months old and the oldest was 15 years and two months. The onset of symptoms was observed before the first month in 16 of the subjects (classified as type Ia), before three months (Ib) in 22 and between 3 and 6 months (Ic) in 13 of them. Regarding the number of copies of *SMN2*, most had two copies, with no significant difference between groups in terms of gene copies. In addition, almost all patients were treated with Nusinersen, and several received a combination of Nusinersen and gene therapy, as shown in Table 1. The treatment received depended mainly on the time of birth, since the evolution of treatments in Spain has been linked to clinical trials and approval of the different drugs by the EMA: In 2015 some children entered clinical trials with Nusinersen; in 2017 and 2018 a compassionate use of said medication was approved for all patients with SMA Type I in Spain without exclusion criteria until its commercialization in 2018 (and its restrictions to access it); in 2019 clinical trials began with Risdiplam, with this medication being approved in 2023 and in 2021 the commercialization of Onasemnogene abeparvovec was approved for those children over two months of age. In addition, 39 subjects received treatment before the age of six months, eight between six and 12 months, two between 12 and 24 months, one patient received treatment at 46 months and another at 108 months.

### 3.2. Clinical and Functional Outcomes

A total of 21.6% (*n* = 11) had tracheostomy (Table 2), however, of these only five needed constant external ventilatory support (≥16 h/d), having initiated such support at a mean age of seven months. A cough assist was used in 42 subjects (82.4%), BiPAP in 37 (72.5%) and 31 used an aspirator (60.8%). As seen in Table 2, more subjects were fed by mouth than by tube/bottle, and of the five subjects who combined feeding by mouth and tube/bottle, one ate very little by mouth, one ate little and two ate quite a lot by mouth. In addition, four had gastroesophageal reflux (7.8%), 14 had had it in the past (27.5%), 32 had none (62.7%) and one did not answer the question (2%). The main orthopedic disorders observed in the cohort of patients were scoliosis and hip subluxation and dislocation, in which case the families were asked if they knew the degrees. Of the 34 patients with scoliosis, 29 were aware of it or had a medical report detailing it, and of the 35 patients who had hip subluxation or dislocation, 24 were able to give the percentage of migration of both hips (Table 2). Moreover, 25 subjects had undergone some surgical intervention (49%), and three of them had suffered fractures, one of them recovered with simple rest and two with external immobilization.

The functional capacities of the patients are presented in Table 3. A subdivision of the sample was proposed in these three groups to test the difference between patients Ia, Ib and Ic, finding that there is no significance regarding belonging to one group or another and the clinical and functional variables of the patients, such as motor milestones acquired, type of feeding or use of mechanical ventilation (*p* > 0.05).

### 3.3. Standardized Assessments

The medians and interquartile ranges of the scales are shown in Table 4. The Chop-Intend scale and Section 2 of the HINE were administered to all subjects, whereas the HMFSE scale was administered to those older than 24 months (*n* = 39) and the RULM to those older than 30 months (*n* = 34). However, of these, seven subjects were uncooperative due to behavior and could not be scored, therefore the RULM scale score was obtained in 27 subjects.

### 3.4. Therapies and Technical Aids

In addition to the information shown in Table 5, 17 subjects performed aquatic therapy, 16 received respiratory physiotherapy, one subject received horse therapy, five received osteopathy sessions and one received music therapy.

In terms of the physiotherapy approach, three families reported receiving therapy based on passive stretching, 21 families received functional and muscle strength training, one family received a family-centered model, seven described their therapy within the framework of the Bobath Concept, one family received Vojta Therapy, and 18 did not fit into any of these categories or reported not knowing exactly what therapy their child was receiving. In addition, the speech therapy approach was classified as myofunctional therapy by 16 families, communication skills according to one family, 12 families reported receiving both communication skills and myofunctional therapy, one received family-centered model, and 15 families did not know how to describe it or did not know what their children did during speech therapy.

The use of orthoses and technical aids showed that 35 children (68.6%) had a standing frame, 40 (78.4%) used DAFOS, 21 used a corset (41.2%), 27 used a dynamic lycra suit (52.9%), 41 used a seat (80.4%), 22 used a walker (43.1%), and 32 had mobility devices (e.g., powered and manual wheelchairs) (62.7%). The standing frames were positioned at a median degree opening of 30° (10) and the age at which mobility devices were introduced was 24 months (18.8).

## 4. Discussion

This study offers data on SMA type I, suggesting that the associated orthopedic, respiratory, and feeding alterations present a new paradigm, which differs from the classical description of the pathology, as well as from its milder versions. Our results show an absence of significant differences between the subgroups divided by age of symptom onset (type Ia, Ib and Ic), which is in contrast to previous reports [13]. This suggests that the factor of age at symptom onset does not influence the functional status of children and that concerning this aspect, SMA type I behaves as a single entity for all the descriptor variables studied in this sample. However, this finding should be taken with caution because although the data were collected on 85% of the population of children with SMA type I in Spain, the sample sizes of each of the subgroups studied are relatively small and this could increase the risk of committing a type II error when comparing between the three groups. Finally, we have found that patients with SMA type I are using technical aids in line with their current functional capacity such as standing frames or walkers.

Observing our sample, the mean age of the participants was 3.7 ± 2.5 years, with only 9.8% (*n* = 5) requiring constant ventilation since an average age of seven months, in contrast to previous data showing higher percentages of need for constant mechanical ventilation [11,35,36]. Regarding feeding, in our sample, 56.9% were able to feed themselves orally, 33.3% ate exclusively by tube/button and 9.8% combined both methods, values lower than those described above in cohorts in which all children with SMA type I older than 12 months required tube/button feeding [9]. In contrast, in a cohort of 122 patients with SMA type II, only 2% required gastrostomy feeding [37].

Due to the severity of the disease and early death, patients with SMA type I did not attend trauma consultations prior to pharmacological treatment [38,39,40,41]. Our study presents a cohort of patients with type I, including the three subgroups but without significant differences between them, observing that 66.7% had scoliosis, in contrast to the data of patients with SMA type II and the more aggressive form of type III, in which 100% of scoliosis was observed at around four years of age [23,40], and at nine years of age in patients with type III [41], showing that the corset was unable to contain the progression of the deformity [23,41]. In the study by Al Amrani et al. [42], seven patients with SMA type I treated with Nusinersen were evaluated, all of whom developed scoliosis in the first year of life. The specific values of scoliosis in our sample ranged from 5° to 100°, in line with what was found in other types of SMA [41,43], between 10° and 113–165° in children with type II and between 10–30° and 92–153° in children with SMA III. The prevalence, severity and progression of scoliosis are much higher in patients with SMA type II than in those with SMA type III [23], therefore, it is likely to be even more so in children with SMA type I, where the probability of requiring scoliosis surgery is 80% [44]. Nonetheless, our cohort revealed different findings, however, it should be considered that at the time of assessment, the children who did not have scoliosis were between 0.33 and 5.33 years old (mean 2.3 years), which may explain the low prevalence.

Regarding hip subluxation or dislocation, there is limited data on patients with SMA type I for the same reason explained above in the case of scoliosis [43,45], although if survival is maintained, one or both hips could dislocate, although they would likely remain asymptomatic [45]. In our sample, 31.4% had hips within normal parameters, 49% presented subluxation and 19.6% had complete dislocation. In the population with SMA type II, approximately 31% have healthy hips, 33–38% have uni- or bilateral subluxation and 11–50% have dislocated hips. In patients with SMA type III, this percentage is 50% with normal hips, 28–29% with uni- or bilateral subluxation and 12–22% with complete dislocation of the hips [43,46]. Again, it is important to note that the mean age of the patients with hips displaying normal parameters was 1.62 years (0.33–3.39), therefore, it is likely that a percentage of them will progress to subluxation or complete dislocation over time. It should be noted that only three of the patients suffered or had suffered from hip pain (6%).

One of the main reasons for the high rates of hip luxation is due to the absence of active movement and weight bearing, along with weakness and laxity of ligaments [43]. The prevention of this problem is presumably determined by standing and walking with or without orthosis [46], which can be seen in our cohort, since 43% used walkers and 68.6% used a standing frame. Along these lines, Townsend et al. conducted a study [47] in which only 13% of type I patients used a standing frame, compared to 68% of type II SMA patients. Our data show that of the children with SMA type I able to walk with a walker or furniture support (*n* = 11), only three had normal hips. The only child able to ambulate without support (albeit with a foot-ankle orthosis) had bilateral hip subluxation. Of these 12 subjects with the motor ability to walk with or without support, only two were not using a static standing frame as part of the treatment to minimize hip migration. The fact that, despite presenting gait with and without support, hip subluxation or dislocation is found, raises the possibility that this orthopedic alteration may respond not only to motor weakness and laxity, but also to the disease phenotype itself. This approach is vitally important to minimize the stress placed on families and caregivers by the progression of hip subluxation despite their efforts to maintain proper positioning that is balanced with dynamic standing and frequent walking.

Probably the most important change in SMA type I patients is the acquisition of motor milestones. Classically, they had difficulty maintaining head control and rarely developed the ability to sit upright [36]. However, in our study, 76.5% acquired head control and 66.7% maintained independent sitting, whereas 30% of patients in the study by Pane et al. [48] were able to sit, and an older sample had begun treatment in a more chronic phase of the disease, finding that in a sample of patients treated before six weeks of age, all patients were able to sit unsupported [49]. Regarding higher motor milestones, in our study, 35.3% were able to stand with support, 7.8% without support; 23.5% were able to walk with one support and 2% (one subject) were able to walk without support, whereas in the study by De Vivo et al. [49], with a very young sample treated pre-symptomatically, 92% walked with support and 88% walked unaided. Regardless of walking ability, it is essential to consider the need for early mobility devices to improve development and cognitive abilities [50,51]. In our sample, 62.7% had manual or powered wheelchairs and the mean age of onset of use was 24 months. In a previous study [38], 75% of the patients had a system for independent mobility, and the mean age that these devices were implemented was 6.4 years. Given that several years have elapsed between the collection of data, it is reasonable to think that the community that cares for people with SMA is aware of the importance and need for implementing independent mobility as soon as possible and is taking these measures.

The evolution of the patients in our sample has led to the use of scales that were classically intended for patients with SMA types II and III, such as the HMFSE, designed for patients with greater motor capacity, or the RULM, which, since it was intended for use after 30 months of age, was focused on patients with SMA types II and III, since type I patients did not survive until that age in most cases. Regarding the median scores obtained in the CHOP-INTEND, the scale with the most data in patients with SMA type I, it is observed that there is no significant difference between the groups by age of symptom onset (Ia, Ib and Ic). The values for the whole sample were 48 points (RI 16 points). These values are much higher than those previously recorded, with a maximum of 33 points, according to the group studied [11], or always being below 22 in the most severe forms of the disease, between 20 and 40 points in the phenotype classified as “typical” and between 39 and 41 in the mildest form of type I [13]. In the sample by Finkel et al. [10], values of between 11 and 32.5 points were obtained, and in that of Pane et al. [48], treated with Nusinersen, a maximum mean of 26.75 was obtained at follow-up.

Thanks to the HINE-2 subscale, which assesses the achievement of motor milestones, it is known that children with SMA type I rarely achieved these milestones [30,36]. Nevertheless, our sample obtained a good score, with a median of 16 (13), bearing in mind that the scale scores from 0 to 26. This is in stark contrast with the cohort of Pechman et al. [24] who obtained results of 2.5 ± 3.3 on average, although this was only after six months of pharmacological treatment and with a group of patients of different ages 24 months after starting treatment [48] in which a mean score of 3.5 ± 4.96 was obtained. Conversely, in a group of children treated pre-symptomatically, a mean score of 3.5 ± 4.96 was obtained [49], and the HINE-2 score was 23.9 (16–26) in the assessment performed at 26.5 months of age, a trend that appears consistent with our sample, in which treatment was initiated relatively early in many cases.

Regarding the HMFSE and RULM scales, it is important to highlight that the scores obtained in our study, 19 points (RI 17 points) and 16 points (RI 8 points), respectively, show the need to include them in the routine care of patients with the new SMA type I phenotype, as previously stated [24]. However, it is important to note that the HMFSE was only administered to 39 children (those older than 24 months) and the RULM was administered to 27 children, using age as the criterion in the study of the scale [34] (30 months or more), and not 24 months as has been proposed by other authors [24], as it was observed that it was a scale in which a longer attention and collaboration time was necessary, with seven subjects who, despite being older than 30 months, did not collaborate.

The strength of this study is that it is the first to be carried out in Spain, with almost the entire population registered with type I SMA. However, it is important to consider the limitations of this study. Firstly, by attempting to obtain current information on the maximum number of patients with SMA type I, we present a sample composed of patients of different ages, who initiated treatment at different times. It is important to note that most patients began treatment before one year of age, one patient began treatment after the age of two years although before the age of five years, and only one patient began treatment after the age of five years. Furthermore, as this is a cross-sectional study, it is not possible to establish a temporal relationship between exposure to the medication and the outcome, understood as the improvement in the variables studied at the level of general health and functionality, which makes it difficult to determine cause and effect. When subdividing into the three groups by age of symptom onset (Ia, Ib and Ic), the sample of each group is small, and stratification by other criteria such as *SMN2* copy number is not possible because more than 88% of the sample had two copies of the gene. Given the low mean age of the sample, there are likely many patients who have not yet developed comorbidities or, conversely, who have not developed higher motor skills due to their young age.

## 5. Conclusions

The current panorama of SMA type I presents a new phenotype which is essential to obtain real-life outcome data to enhance our understanding of this disorder. According to the results of the study, it is currently a different entity to classic SMA type I and also to SMA type II and III, with which, although it may now share functional capacities, it presents differences in terms of respiratory, feeding or orthopedic alterations. It is especially important to consider that children with SMA type I are currently able to sit and many can even stand and walk, bearing in mind the high frequency of scoliosis and subluxation and dislocation of the hips, as well as other health problems. In addition, the age of symptom onset did not lead to differences in the clinical and functional status of patients with SMA type I, and they behaved as a single group.

## Figures and Tables

**Table 1:** Demographic characteristics of participants *.

	Ia	Ib	Ic	Total
Number (%)	16 (31.4)	22 (43.1)	13 (25.5)	51 (100)
Sex, *n* (%) of children				
Male	10 (62.5)	12 (54.5)	9 (69.2)	31 (60.8)
Age of symptom onset, months, median (IR) ^†^	0.2 (0.71)	2 (1.5)	4 (1)	2 (2.64)
Age at beginning of treatment, months, median (IR) ^†^	2 (3.35)	4 (2.48)	7 (8.25)	4.5 (4)
Age at time of assessment, years, median (IR)				
	3.6 (2.69)	3.2 (3.17)	3.8 (2.99)	3.6 (2.6)
*SMN2* Copies, *n* (%)				
1	0	1 (4.5)	0	1 (2)
2	15 (93.8)	21 (95.5)	9 (69.2)	45 (88.2)
3	0	0	2 (15.4)	2 (3.9)
Unknown	1 (6.3)	0	2 (15.4)	3 (5.9)
Treatment, *n* (%)				
Nusinersen	9 (56.3)	20 (90.9)	10 (76.9)	39 (76.5)
Risdiplam	1 (6.3)	0	1 (7.7)	2 (3.9)
Onasemnogene abeparvovec	1 (6.3)	0	1 (7.7)	2 (3.9)
Onasemnogene abeparvovec + Nusinersen	5 (31.3)	2 (9.1)	1 (7.7)	8 (15.7)

*SMN2*: Survival Motor Neuron 2. IR: Interquartile Range. * No significant differences between symptom onset subgroups, *p* > 0.05. ^†^ Significant differences between symptom onset subgroups, *p* < 0.001.

**Table 2:** Clinical characteristics of participants *.

	Ia	Ib	Ic	Total
Tracheostomy, *n* (%)	5 (31.3)	4 (18.2)	2 (15.4)	11 (21.6)
Ventilation ≥ 16 h/día, *n* (%)	4 (25%)	0	1 (7.69)	5 (9.8)
Feeding, *n* (%)				
Mouth	8 (50)	13 (59.1)	8 (61.5)	29 (56.9)
Feeding tube/Button	6 (37.5)	8 (36.4)	3 (23.1)	17 (33.3)
Mixt	2 (12.5)	1 (4.5)	2 (15.4)	5 (9.8)
Orthopedic disorders				
Scoliosis *n* (%)	14 (87.5)	12 (54.5)	8 (61.5)	34 (66.7)
Degrees of Scoliosis, median (IR)	40 (38)	30 (12)	33.5 (69)	30 (23)
(*n*)	(*n* = 12)	(*n* = 11)	(*n* = 6)	(*n* = 29)
Hip subluxation/dislocation *n* (%)	11 (68.8)	14 (63.6)	10 (76.9)	35 (68.6)
Migration left hip, median (IR)	72 (70)	52.5 (80)	34 (75)	50 (72)
(*n*)	(*n* = 10)	(*n* = 8)	(*n* = 6)	(*n* = 24)
Migration right hip, median (IR)	68 (71)	52.5 (79)	54.5 (63)	52.5 (72)
(*n*)	(*n* = 10)	(*n* = 8)	(*n* = 6)	(*n* = 24)

IR: Interquartile Range. * No significant differences between symptom onset subgroups, *p* > 0.05.

**Table 3:** Functional characteristics of patients *.

	Ia	Ib	Ic	Total
Head control, *n* (%)	10 (62.5)	18 (81.8)	11 (84.6)	39 (76.5)
Independent sitting, *n* (%)	10 (62.5)	14 (63.6)	10 (76.9)	34 (66.7)
Standing with support, *n* (%)	5 (31.3)	11 (50)	2 (15.4)	18 (35.3)
Standing without support, *n* (%)	1 (6.3)	3 (13.6)	0	4 (7.8)
Walking with support, *n* (%)	3 (18.8)	8 (36.4)	1 (7.7)	12 (23.5)
Walking unsupported, *n* (%)	1 (6.3)	0	0	1 (2)

* No significant differences between symptom onset subgroups, *p* > 0.05.

**Table 4:** Scoring of scales by subclassification group *.

	Ia	Ib	Ic	Total
CHOP-INTEND, median (IR)	47 (16)	51 (17)	48 (12)	48 (16)
HINE-2, median (IR)	16 (14)	17.5 (11)	15 (9)	16 (13)
Hammersmith, median (IR) (*n*)	19 (22) (*n* = 13)	22 (20) (*n* = 16)	11.5 (18) (*n* = 10)	19 (17) (*n* = 39)
RULM, median (IR) (*n*)	19 (19) (*n* = 8)	16 (8) (*n* = 13)	15.5 (19) (*n* = 6)	16 (8) (*n* = 27)

CHOP INTEND: The Children’s Hospital of Philadelphia Infant Test for Neuromuscular Disorders; HINE-2: Hammersmith Infant Neurological Examination-2; HFMSE: Hammersmith Functional Motor Scale Expanded; RULM: Revised Upper Limb Module. IR: Inter Quartile Range. * No significant differences between symptom onset subgroups, *p* > 0.05.

**Table 5:** Therapies received.

	Physiotherapy	Speech Therapy	Occupational Therapy	Psychology
*n* (%)	51 (100)	45 (88.2)	13 (25.5)	8 (15.7)
Frequency (h/s), median (IR)	2.5 (1.5)	1.5 (1.23)	0.75 (0.5)	0.75 (0.19)

h/s: Hours per week. IR: Interquartile Range.

## Data Availability

The datasets generated and/or analyzed during the current study are not publicly available due to ethics restrictions but are available from the corresponding author on reasonable request.

## Supplementary Materials

The following supporting information can be downloaded at: https://www.mdpi.com/article/10.3390/children10050892/s1, Table S1: STROBE checklist.
